# Pharmacological Characterization of Inositol 1,4,5-tris Phosphate Receptors in Human Platelet Membranes

**DOI:** 10.1155/2009/618586

**Published:** 2009-10-12

**Authors:** Yogesh Dwivedi, Ghanshyam N. Pandey

**Affiliations:** Department of Psychiatry, The Psychiatric Institute, University of Illinois at Chicago, Chicago, IL 60612, USA

## Abstract

The phosphatidylinositol (PI) hydrolysis signaling system has been shown to be altered in platelets of depressed and schizophrenic subjects. Inositol (1,4,5) trisphosphate (Ins(1,4,5)P_3_), an integral component of the PI signaling system, mobilizes Ca^2+^ by activating Ins(1,4,5)P_3_ receptors. To eventually investigate the role of Ins(1,4,5)P_3_ receptors in depression and other mental disorders, we characterized [^3^H]Ins(1,4,5)P_3_ binding sites in crude platelet membranes prepared from small amounts of blood obtained from healthy human control subjects. We found a single, saturable binding site for [^3^H]Ins(1,4,5)P_3_ to crude platelet membranes, which is time dependent and modulated by pH, inositol phosphates, and heparin. Since cyclic adenosine monophosphate (cAMP) and Ca^2+^ have been shown to be important modulators in Ins(1,4,5)P_3_ receptors, in the present study we also determined the effects of various concentrations of CaCI_2_ and forskolin on Ins(1,4,5)P_3_ binding to platelet membranes. CaCI_2_ modulated [^3^H]Ins(1,4,5)P_3_ binding sites in a biphasic manner: at lower concentrations it inhibited [^3^H]Ins(1,4,5)P_3_ binding, whereas at higher concentrations, it stimulated [^3^H]Ins(1,4,5)P_3_ binding. On the other hand, forskolin inhibited [^3^H]Ins(1,4,5)P_3_ binding. Our results thus suggest that the pharmacological characteristics of [^3^H]Ins(1,4,5)P_3_ binding to crude platelet membranes are similar to that of Ins(1,4,5)P_3_ receptors; and that both Ca^2+^ and cAMP modulate [^3^H]Ins(1,4,5)P_3_ binding in crude platelet membranes.

## 1. Introduction

Agonist-stimulated activation of cell surface receptors, such as 5HT_2A_, 5HT_2C_, *α* adrenergic, and muscarinic receptors, leads to the hydrolysis of phosphatidylinositol 4,5-bisphosphate by stimulating phospholipase C (PLC) and subsequently generating two second messengers: diacylglycerol (DG) and inositol 1,4,5-trisphosphate (Ins(1,4,5)P_3_). DG activates protein kinase C (PKC) [1], while Ins(1,4,5)P_3_ triggers the release of Ca^2+^ from intracellular sources by interacting with Ins(1,4,5)P_3_ receptors [2–4]. Ins(1,4,5)P_3_ receptors have been identified and characterized in both central and peripheral tissues, such as the brain [5], the hepatic plasma membranes [6], smooth muscle cells [7, 8], rat cerebral and bovine adrenocortical membranes [9], and the rat cerebellum [10]. Cloning studies in various tissues show the existence of three types (I, II, and III) of Ins(1,4,5)P_3_ receptors [11–13]. All are believed to act as Ca^2+^ channels [4, 14].

Similar to other cell types, Ins(1,4,5)P_3_ in platelets also functions as a second messenger and mobilizes calcium (Ca^2+^) by activating the Ins(1,4,5)P_3_ receptor site [15, 16], which plays an important role in platelet responses involved in homeostasis and thrombosis. Platelets offer a suitable peripheral model for studying abnormalities in neurotransmitter receptors and receptor-mediated second messenger systems such as adenylyl cyclase-cyclic adenosine monophosphate (cAMP) and the phosphatidyl inositol (PI) hydrolysis signaling system. Some reports indicate that the PI signaling system is altered in the platelets of depressed and schizophrenic subjects. Kaiya et al. [17] showed an increase in DG in the platelets of schizophrenic subjects and proposed that this increase may cause a decrease in Ins(1,4,5)P_3_/Ca^2+^ function. Also Mikuni et al. [18] reported an increase in 5HT-induced accumulation of inositol phosphate-1 (IP_1_) in the platelets of depressed patients compared to control subjects. We also observed that thrombin-stimulated IP_1_ receptors formation was significantly greater in depressed patients compared to control subjects [19]. Since the above-mentioned studies indicate abnormalities in the PI signaling system in depression, it is quite possible that these abnormalities may also be associated with alterations in Ins(1,4,5)P_3_ receptors. Therefore, it is important to examine Ins(1,4,5)P_3_ receptors in the platelets of these subjects. So far, the role of Ins(1,4,5)P_3_ receptors in platelets of patients with mental disorders has not been studied. To eventually examine if Ins(1,4,5)P_3_ receptors are altered in depressed subjects and patients with other mental disorders, we characterized Ins(1,4,5)P_3_ receptors in crude platelet membranes obtained from normal human control subjects. Although Ins(1,4,5)P_3_ receptors have been characterized in purified intracellular human platelet membranes rich in dense tubular systems [20], for clinical studies it is important to examine if Ins(1,4,5)P_3_ receptors can be studied in crude membranes since it is not feasible to obtain large amounts of blood from patient populations.

Ca^2+^ and cAMP are two potent modulators in Ins(1,4,5)P_3_ receptors in various tissues (5,21,22,23). Depending on its concentration, Ca^2+^ has been shown to alter [^3^H]Ins(1,4,5)P_3_ binding in various tissues (5,24,25). To further investigate if Ca^2+^ affects [^3^H]Ins(1,4,5)P_3_ binding, we studied the effects of various concentrations of CaCl_2_ on [^3^H]Ins(1,4,5)P_3_ binding to crude platelet membranes.

cAMP is known to release Ca^2+^ from permeabilized platelets. cAMP also phosphorylates Ins(1,4,5)P_3_ receptors via cAMP-dependent protein kinase A (PKA) [21]. However, whether cAMP affects [^3^H]Ins(1,4,5)P_3_ binding to platelet membranes, has not yet been studied. To determine if cAMP has any effect on Ins(1,4,5)P_3_ receptors, we investigated the effect of forskolin, which is known to release endogenous cAMP, on [^3^H]Ins(1,4,5)P_3_ binding to crude platelet membranes.

 Findings of the present study suggest that the pharmacological characteristics of [^3^H]Ins(1,4,5)P_3_ binding to crude platelet membranes are similar to that of Ins(1,4,5)P_3_ receptors; and that both Ca^2+^ and cAMP modulate [^3^H]Ins(1,4,5)P_3_ binding in crude platelet membranes. With these properties, IP3 receptors can be successfully measured in platelet membranes that can be used to as diagnostic marker for major mental illnesses, including major depression, where abnormalities in PI signaling system have been reported.

## 2. Materials and Methods

### 2.1. Chemicals

D-myo-[^3^H]inositol 1,4,5-trisphosphate (specific activity 21 Ci/mmol) was obtained from New England Nuclear (Boston, MA). D-myo-inositol 1,4,5-trisphosphate, L-myo-inositol 1,4,5-trisphosphate, D-myo-inositol 2,4,5-trisphosphate, L-*α*-glycerophosphoinositol 4,5-bisphosphate (GPIP_2_), heparin sulfate, and forskolin were purchased from Sigma Chemical Co. (St. Louis, MO). All other reagents were of analytical grade and were obtained from Sigma Chemical Co.

### 2.2. Preparation of Platelet Membranes

Blood (10 to 20 mL) was drawn from healthy normal human subjects into a tube containing 3.8% (w/v) sodium citrate. The blood was centrifuged immediately at 210 × g for 10 minutes at 4°C to obtain platelet-rich plasma (PRP), which was centrifuged at 4000 × g for 10 minutes at 4°C. The platelet pellet thus obtained was homogenized by polytron (at #7 setting) for 30 seconds in a homogenizing buffer containing 50 mM Tris-HCl, pH 7.7; 1 mM ethylene diamine N′, N′, N′, N′-tetraacetic acid (EDTA); and 2 mM 2-mercaptoethanol. The homogenate was centrifuged at 40,000 × g for 15 minutes at 4°C. The supernatant was discarded and the pellet was homogenized once again in the homogenizing buffer and centrifuged as described above. The resulting pellet was resuspended in a buffer containing 50 mM Tris-HCl, pH 8.5; 1 mM EDTA; and 1 mM 2-mercaptoethanol. This fraction was used for the [^3^H]Ins(1,4,5)P_3_ binding assay. To examine the effect of CaCl_2_, the binding assay was performed in presence or absence of EDTA, as described below.

### 2.3. [^3^H]Ins(1,4,5)P_3_ Binding Assay

The binding of [^3^H]Ins(1,4,5)P_3_ to crude human platelet membranes was carried out in duplicate. The incubation medium contained incubation buffer (50 mM Tris-HCl, pH 8.5; 1 mM 2-mercaptoethanol; 1 mM EDTA), [^3^H]Ins(1,4,5)P_3_ (specific activity 21 Ci/mmol) ranging from 10 to 100 nM (six different concentrations), and 40 *μ*L of platelet membrane suspension in a total volume of 100 *μ*L. Nonspecific binding was determined in the presence of 10 *μ*M Ins(1,4,5)P_3_. The incubation was performed at 4°C for 10 minutes and rapidly terminated by the addition of 5 mL of cold buffer (50 mM Tris-HCl, pH 7.7; 1 mM EDTA; and 0.1% (w/v) bovine serum albumin) and filtration through Whatman GF/B filters. The filter-bound radioactivity was analyzed by a liquid scintillation counter. Specific binding was defined as the difference between the total binding and the binding observed in the presence of D-Ins(1,4,5)P_3_. The maximum number of binding sites (*B*_max_) and the apparent dissociation constant (*K*_*d*_) were computed by Scatchard analysis using the EBDA program [22]. Protein was determined by the method of Lowry et al. [23]. IC_50_ (concentration of agents necessary to inhibit half of the specific Ins(1,4,5)P_3_ binding) values of different agents (D-Ins(1,4,5)P_3_; D-Ins(2,4,5)P_3_; L-Ins(1,4,5)P_3_; GPIP_2_; and heparin) for the inhibition of specific binding were determined by log probit analysis.

 To examine the pH-dependence of [^3^H]Ins(1,4,5)P_3_ binding and time course for specific binding of [^3^H]Ins(1,4,5)P_3_, 20 nM of [^3^H]Ins(1,4,5)P_3_ was used.

### 2.4. Determination of the Effect of *CaCl*_2_ on [^3^H]Ins(1,4,5)P_3_ Binding

The effects of CaCl_2_ were studied in the presence and in the absence of EDTA. Platelet membranes (100 *μ*g protein) were incubated with CaCl_2_ (0.5 to 30 mM) in a buffer containing 50 mM Tris-HCl, pH 8.5; 1 mM 2 mercaptoethanol; and 20 mM [^3^H]Ins(1,4,5)P_3_. EDTA (1 mM) was added to the incubation medium in which the effect of CaCl_2_ was studied in the presence of EDTA. The incubation was carried out at 4°C for 10 minutes.

### 2.5. Determination of the Effect of Forskolin on [^3^H]Ins(1,4,5)P_3_ Binding

Platelet protein samples (100 *μ*g) were incubated with forskolin (10^−4^ to 10^−6^ M) in a buffer containing 50 mM Tris-HCl, pH 8.5; 1 mM EDTA; and 1 mM 2-mercaptoethanol. The incubation was carried out at 4°C for 10 minutes.

## 3. Results

### 3.1. pH-Dependence of [^3^H]Ins(1,4,5)P_3_ Binding to Platelet Membranes

**Figure 1:**
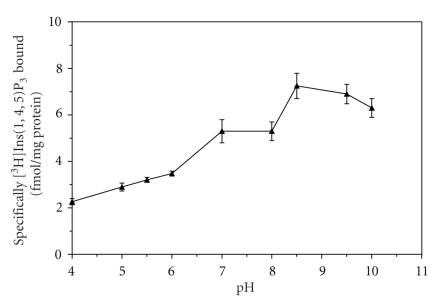
The effect of pH on [^3^H]Ins(1,4,5)P_3_ binding to human platelet membranes. Binding at different pH values was performed as described in Section 2. The reaction mixture contained 20 nM [^3^H]Ins(1,4,5)P_3_ and approximately 100 *μ*g protein. Nonspecific binding was determined in the presence of 10 *μ*M D-Ins(1,4,5)P_3_. Incubations were carried out at 4°C for 10 minutes. Each point represents the mean value of two experiments performed in duplicate.

The results presented in Figure 1 show that [^3^H]Ins(1,4,5)P_3_ binding to crude platelet membranes is pH dependent. We measured specific binding of [^3^H]Ins(1,4,5)P_3_ between pH 4 and 10. Specific binding of [^3^H]Ins(1,4,5)P_3_ was relatively low at acidic pH (pH 4.0 to 6.0), and increased by 60% as the pH approached 7.0. Specific binding was stable from pH 7.0 to 8.0, but it increased further between pH 8.0 to 8.5, and thereafter declined.

### 3.2. Time Course for Specific Binding of [^3^H]Ins(1,4,5)P_3_ to Platelet Membranes

**Figure 2:**
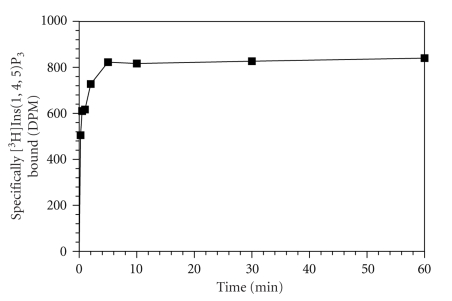
Time course of [^3^H]Ins(1,4,5)P_3_ binding to human platelet membranes. Platelet membranes (100 *μ*g/protein) were incubated at 4°C for different time intervals in the presence of 20 nM [^3^H]Ins(1,4,5)P_3_. Nonspecific binding was estimated in the presence of 10 *μ*M D-Ins(1,4,5)P_3_. The incubations were rapidly terminated by vacuum filtration at different time points as shown in the figure. A single representative experiment is shown for duplicate determinations.

We determined the time course for [^3^H]Ins(1,4,5)P_3_ binding to crude platelet membranes from 15 seconds up to 60 minutes. As shown in Figure 2, the binding of [^3^H]Ins(1,4,5)P_3_ to Ins(1,4,5)P_3_ receptors was very rapid. At 15 seconds the specific binding was very low but it reached equilibrium within 5 minutes. After that, specific binding remained constant up to 60 minutes.

### 3.3. Saturation Isotherm of [^3^H]Ins(1,4,5)P_3_ Binding to Platelet Membranes

**Figure 3:**
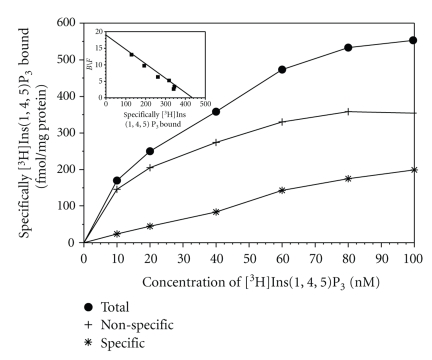
Saturation isotherm of [^3^H]Ins(1,4,5)P_3_ binding to human platelet membranes. Each point is the mean of duplicate determination. Binding assays were carried out as described in Section 2. Nonspecific binding was determined in the presence of 10 *μ*M D-Ins(1,4,5)P_3_. A Scatchard plot of [^3^H]Ins(1,4,5)P_3_ binding is shown in the inset. *B* = [^3^H]Ins(1,4,5)P_3_ specifically-bound (fmol/mg protein), B/F = the ratio of specifically-bound to free ligand in fmol of Ins(1,4,5)P_3_ (fmol/mg protein × nM). For this particular experiment, binding indices are *B*_max_ = 427.56 fmol/mg protein *K*_*d*_ = 21.73 nM; and the correlation coefficient (*r*) = 0.98.

The maximum number of binding sites (*B*_max_) and the apparent dissociation constant (*K*_*d*_) in crude platelet membranes were determined by using different concentrations of [^3^H]Ins(1,4,5)P_3_. Nonspecific binding was determined in the presence of 10 *μ*M D-Ins(1,4,5)P_3_. Initially, we performed the experiments using 0.1 to 100 nM [^3^H]Ins(1,4,5)P_3_. We observed that at lower concentrations of [^3^H]Ins(1,4,5)P_3_ (0.1 to 10 nM), the displacement was too low to draw the Scatchard plot. A concentration range of 10 to 100 nM [^3^H]Ins(1,4,5)P_3_, however, showed a specific binding of 80% to 50% depending upon the concentration of [^3^H]Ins(1,4,5)P_3_. Figure 3 represents a typical saturation isotherm and a Scatchard plot (inset) of [^3^H]Ins(1,4,5)P_3_ binding to platelet membranes. Specific binding is saturable between 80 to 100 nM [^3^H]Ins(1,4,5)P_3_. Nonspecific binding is nonsaturable and linear with a concentration of 10 to 100 mM [^3^H]Ins(1,4,5)P_3_. The Scatchard plot indicates a single class of binding site. The means of B_max_ and K_d_ of five independent experiments performed in duplicate were found to be 427.77 ± 56.67 fmol/mg proteins and 22.09 ± 2.34 nM, respectively.

### 3.4. Specificity of [^3^H]Ins(1,4,5)P_3_ Binding

**Figure 4:**
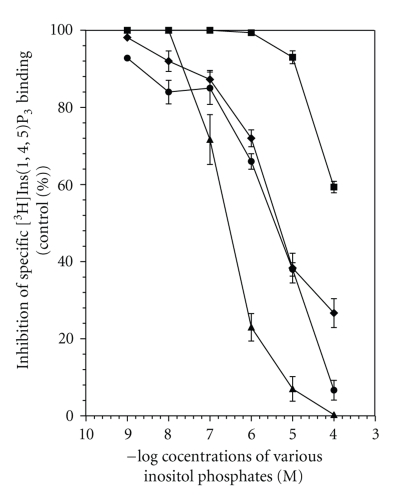
Displacement curve for the inhibition of [^3^H]Ins(1,4,5)P_3_ binding to human platelet membranes by inositol phosphates. Platelet membranes were incubated with 20 nM [^3^H]Ins(1,4,5)P_3_ in the presence of increasing concentrations of D-Ins(1,4,5)P_3_ (*▲*), D-Ins(2,4,5)P_3_ (*●*), GPIP_2_ (*♦*), and L-Ins(1,4,5)P_3_ (■) at 4°C for 10 minutes. The data are the mean ± S.E.M. of three independent experiments, each run in duplicate. The IC50 values were calculated using the displacement curve (Table 1).

**Figure 5:**
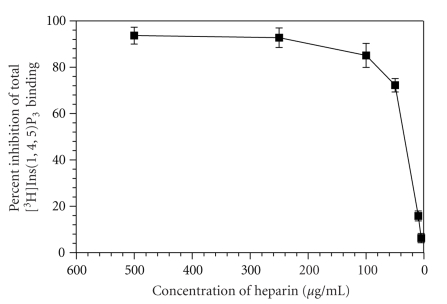
Inhibition of [^3^H]Ins(1,4,5)P_3_ binding to human platelet membranes by heparin. Experimental conditions are similar to those described in Figure 4. The points represent the mean ± S.E.M. of three independent experiments, each run in duplicate.

The pharmacological characterization of Ins(1,4,5)P_3_ receptors was carried out using different agents known to inhibit [^3^H]Ins(1,4,5)P_3_ binding. A displacement curve of [^3^H]Ins(1,4,5)P_3_ binding with different concentrations of inositol phosphates is shown in Figure 4. Of the various inositol phosphates, D-Ins(1,4,5)P_3_ was found to be the most potent inhibitor of [^3^H]Ins(1,4,5)P_3_ binding, with an IC_50_ value of 0.3 *μ*M. Next in order were D-Ins(2,4,5)P_3_ (IC_50_ = 1.9 *μ*M), GPIP_2_ (IC_50_ = 4.97 *μ*M), and L-Ins(1,4,5)P_3_ (IC_50_ = 354 *μ*M). Heparin acts as an antagonist on the Ins(1,4,5)P_3_ receptor and inhibited [^3^H]Ins(1,4,5)P_3_ binding in a concentration-dependent manner (5 to 500 *μ*g/mL), with an IC_50_ value of 36.79 ± 6.0 l *μ*g/mL (Table 2 and Figure 5).

### 3.5. Effects of CaCl_2_ on [^3^H]Ins(1,4,5)P_3_ Binding to Platelet Membranes

**Figure 6:**
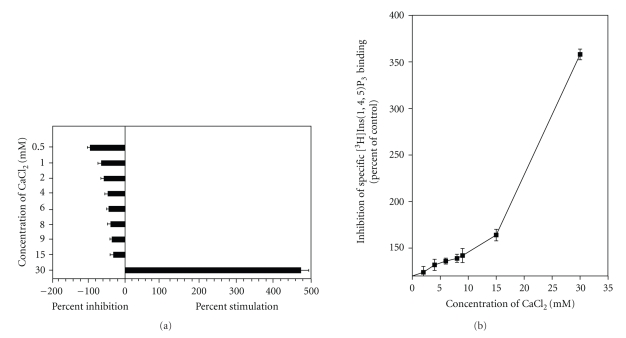
Effects of CaCl_2_ on [^3^H]Ins(1,4,5)P_3_ binding to human platelet membranes in the presence (a) and absence (b) of EDTA. Platelet membranes were incubated with increasing concentrations of CaCl_2_ in the presence of 20 nM [^3^H]Ins(1,4,5)P_3_ in a buffer containing 50 mM Tris-HCl, pH 8.4; 1 mM EDTA; and 1 mM 2-mercaptoethanol at 4°C for 10 minutes. The data represent the inhibition or stimulation of [^3^H]Ins(1,4,5)P_3_ binding to platelet membranes. The points represent the mean ± S.E.M. of three independent experiments, each run in duplicate.

Ca^2+^ has been shown to be a potent modulator of [^3^H]Ins(1,4,5)P_3_ receptors. In the present investigation, we determined the effects of CaCl_2_ on [^3^H]Ins(1,4,5)P_3_ binding to crude platelet membranes in the presence and in the absence of 1 mM EDTA. In the presence of EDTA, CaCl_2_ inhibited [^3^H]Ins(1,4,5)P_3_ binding in a linear fashion depending upon its concentration. At a lower concentration (0.5 mM) the degree of inhibition was maximum (68%), while at a higher concentration (15 mM) the inhibition was very low (5%). At concentrations above 15 mM, however, CaCl_2_ stimulated [^3^H]Ins(1,4,5)P_3_ binding, and at 30 mM CaCl_2_, a four- to fivefold increase in [^3^H]Ins(1,4,5)P_3_ binding was observed (Figure 6). In another set of experiments, we observed the effects of CaCl_2_ in the absence of EDTA. The results, shown in Figure 6(b), demonstrate that CaCl_2_ increased [^3^H]Ins(1,4,5)P_3_ binding to platelet membranes between 24% to 64% depending on the concentration (2 to 15 mM). At a CaCl_2_ concentration of 30 mM, the stimulation of [^3^H]Ins(1,4,5)P_3_ binding to crude platelet membranes was nearly the same (fourfold) as that observed in the presence of 1 mM EDTA.

### 3.6. Effects of In-Vitro Addition of Forskolin on [^3^H]Ins(1,4,5)P_3_ Binding in Platelet Membranes

**Figure 7:**
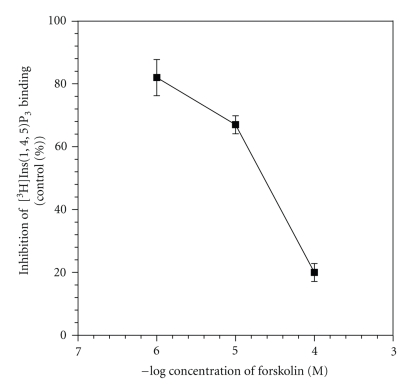
Inhibitory effect of forskolin on [^3^H]Ins(1,4,5)P_3_ binding to human platelet membranes. The experiments were performed in an assay medium (50 mM Tris HC1, pH 8.4; 1 mM EDTA; 1 mM 2-mercaptoethanol) containing 30 mM CaCl_2_ 20 nM [^3^H]Ins(1,4,5)P_3_, and increasing concentrations of forskolin at 4°C for 10 minutes. The data are the mean ± S.E.M. of three independent experiments, each carried out in duplicate.

Since cAMP has been shown to inhibit Ca^2+^ release from permealized platelets, and there is indirect evidence which shows that cAMP causes the phosphorylation of Ins(1,4,5)P_3_ receptors by stimulating PKA, we studied the effects of forskolin on [^3^H]Ins(1,4,5)P_3_ binding to platelet membranes. Forskolin is a potent stimulator of adenylyl cyclase in platelets and thus increases cAMP levels of hydrolyzing adenosine trisphosphate (ATP). We added different concentrations of forskolin in vitro, and the results, given in Figure 7, show that forskolin inhibited [^3^H]Ins(1,4,5)P_3_ binding in a concentration-dependent manner. At a concentration of 10^−4^ M, inhibition was about 80%, whereas at lower concentrations (10^−5^ to 10^−6^ M), forskolin inhibited Ins(1,4,5)P_3_ binding by 20% to 30%.

## 4. Discussion

The pharmacological properties of Ins(1,4,5)P_3_ receptors have been characterized in several tissues including the brain [5, 8]. In an earlier study, Varney et al. [24] observed a single [^3^H]Ins(1,4,5)P_3_ binding site in crude platelet membranes; however, they did not fully characterize Ins(1,4,5)P_3_ receptors in this fraction of platelets. To investigate if Ins(1,4,5)P_3_ receptors in crude platelet membranes possess similar pharmacological properties as observed in other tissues, we characterized [^3^H]Ins(1,4,5)P_3_binding sites in crude platelet membranes obtained from normal human control subjects. We observed a single, saturable binding site of [^3^H]Ins(1,4,5)P_3_ in crude platelet membranes, with a binding capacity of 427.77 fmol/mg protein and an affinity of 22.09 nM. The binding of [^3^H]Ins(1,4,5)P_3_ was pH- and time dependent. The optimum pH was found to be 8.5, with a steep change occurring in the pH range of 4 to 9. Time-course experiments revealed that maximum binding occurred at 2 minutes and remained stable up to 60 minutes. We determined IC_50_ values of different inositol phosphates in crude platelets membranes. D-Ins(1,4,5)P_3_ was found to be the most potent, with an IC_50_ value of 0.3 *μ*M. Heparin is a competitive antagonist of the Ins(1,4,5)P_3_ receptor [5]. In the present study, heparin inhibited [^3^H]Ins(1,4,5)P_3_ binding in a concentration-dependent manner, with an IC_50_ value of 36.79 *μ*g/mL. These pharmacological properties of Ins(1,4,5)P_3_ receptors in crude platelet membranes were found to be similar to those reported in other tissues [5, 8]. Earlier, Hwang [20] characterized [^3^H]Ins(1,4,5)P_3_ receptors in purified human platelet membranes rich in dense tubular systems. In this fraction of platelet membranes, Hwang reported two binding sites of [^3^H]Ins(1,4,5)P_3_, one with low affinity, and another with high affinity; however in our study, we observed only one binding site in crude platelet membranes, which is very similar to that reported by Varney et al. [24]. One binding site has also been reported in other tissues [6, 10, 25]. It is quite possible that the explanation of the single binding site observed by us and Varney et al. in crude platelet membranes and the two binding sites observed by Hwang in purified platelet membranes may be the differences in the preparation of platelet membranes. The pH- and the time-course profiles observed in the present study are similar to those reported in other tissues, including platelets [5, 7, 9, 20].

In continuation of our study, we further investigated the effects of agents that are known to modulate Ins(1,4,5)P_3_ receptors. One such agent is Ca^2+^, which has been shown to affect [^3^H]Ins(1,4,5)P_3_ binding in most tissues [5, 25, 26]. In the present investigation, we studied the effects of various concentrations of CaCl_2_ on [^3^H]Ins(1,4,5)P_3_ binding to crude platelet membranes. Since EDTA is known to chelate Ca^2+^ and our incubation medium contained 1 mM EDTA, we studied the effects of CaCl_2_ in the presence and in the absence of EDTA. We observed that CaCl_2_ stimulated [^3^H]Ins(1,4,5)P_3_ binding in a concentration-dependent manner (2 to 15 mM), and at a concentration of 30 mM, the stimulation was four- to fivefold. In the presence of EDTA, however, CaCl_2_ inhibited [^3^H]Ins(1,4,5)P_3_ binding. We observed maximum inhibition at 0.5 mM, and the degree of inhibition decreased as the concentration of CaCl_2_ increased (0.5 to 15 mM); and at a concentration of 30 mM, CaCl_2_ stimulated [^3^H]Ins(1,4,5)P_3_ binding, the degree of stimulation being similar to that observed in the absence of EDTA. These results suggest a biphasic response of CaCl_2_ on [^3^H]Ins(1,4,5)P_3_ binding to platelet membranes.

The mechanism by which Ca^2+^ inhibits [^3^H]Ins(1,4,5)P_3_ binding in platelets is presently unclear. Delfert et al. [27] reported an inhibitory effect in the release of Ca^2+^ from the endoplasmic reticulum in the presence of free Ca^2+^. It is possible that Ca^2+^ itself regulates the further release of Ca^2+^ by inhibiting Ins(1,4,5)P_3_ binding. Danoff et al. [28] hypothesized that the inhibitory effect of Ca^2+^ is mediated by a protein called calmedin in the cerebral membranes of rats; while Mignery et al. [29] suggested that Ca^2+^-induced inhibition is mediated by Ca^2+^-activated PLC, which produces additional Ins(1,4,5)P_3_, with an apparent decrease in [^3^H]Ins(1,4,5)P_3_ binding. Whether this mechanism exists in platelets is not known at the present time.

Our studies also indicate that EDTA markedly alters the effect of CaCl_2_ on [^3^H]Ins(1,4,5)P_3_ binding. There is a possibility that 1 mM EDTA chelated most of the Ca^2+^, leaving a micromolar concentration of Ca^2+^, which was able to inhibit [^3^H]Ins(1,4,5)P_3_ binding, since it has been shown that inhibition of [^3^H]Ins(1,4,5)P_3_ binding occurs at micromolar concentrations of Ca^2+^ [30]. However, when we increased the concentration of CaCl_2_ to 30 mM, there was not enough EDTA present in the medium to chelate Ca^2+^, and the concentration of Ca^2+^ present in the medium was high enough to stimulate [^3^H]Ins(1,4,5)P_3_ binding. In the absence of EDTA, however, CaCl_2_ stimulated [^3^H]Ins(1,4,5)P_3_ binding. The mechanism by which CaCl_2_ potentiated the binding is not known at this present time and needs further study. Nonetheless, this study suggests that CaCl_2_ modulates [^3^H]Ins(1,4,5)P_3_ binding in platelets.

Another important modulator in Ins(1,4,5)P_3_ receptors is cAMP, which has been shown to release Ca^2+^ from platelet membranes and is involved in phosphorylation of [^3^H]Ins(1,4,5)P_3_ receptors [31, 32]. To investigate the effects of cAMP on [^3^H]Ins(1,4,5)P_3_ binding, we added forskolin in vitro to the assay medium. Forskolin is known to act directly on adenylyl cyclase, thereby generating endogenous cAMP. We found that forskolin inhibited [^3^H]Ins(1,4,5)P_3_ binding in platelets in a concentration-dependent manner. The mechanism by which cAMP inhibits [^3^H]Ins(1,4,5)P_3_ binding is not clear at this present time. It is quite possible that the inhibition observed in [^3^H]Ins(1,4,5)P_3_ binding to platelet membranes by forskolin might be due to the phosphorylation of Ins(1,4,5)P_3_ receptors by cAMP-dependent PKA [31, 32]. The possibility that forskolin acts directly on [^3^H]Ins(1,4,5)P_3_ binding cannot be ruled out, however.

 In summary, our study shows a single, saturable binding site for [^3^H]Ins(1,4,5)P_3_ in crude platelet membranes, which is time dependent and modulated by pH, inositol phosphates, heparin, Ca^2+^, and cAMP. Although there is a minor difference between the results obtained in crude platelet membranes in this study as compared to the results in purified platelet membranes, the pharmacological characteristics of [^3^H]Ins(1,4,5)P_3_ binding to crude platelet membranes are similar to the pharmacological properties of Ins(1,4,5)P_3_ receptors in other tissues, including platelets. These results are important, especially considering that due to the difficulty of obtaining large-enough samples of blood from patients, preparation of purified platelet membranes is not feasible for clinical research. Since the preparation of crude platelet membranes is convenient and requires a smaller amount of blood, this procedure can be utilized to study the role of Ins(1,4,5)P_3_ receptors in depression and other mental disorders, such as schizophrenia or bipolar disorders. Even, within a specific diagnosis, measuring IP3 receptors may be helpful in distinguishing subtypes of mental illness. For example, it will be interesting to examine whether IP3 receptors are altered in a subset of depressed patients. In this regard, the prime example is protein kinase A, which has been shown to be altered in a subtype of depressed patients, that is, melancholic depressed patients or patients who committed suicide [33, 34]. Thus, measuring IP3 receptors in blood cells may lead to the development of novel interventions that could target specific points of vulnerability.

## Figures and Tables

**Table 1:** Apparent maximum binding sites (*B*_max_) and dissociation constants (*K*_*d*_) of [^3^H]Ins(1,4,5)P_3_ binding to human platelet membranes. Saturation analysis of Ins(1,4,5)P_3_ binding sites was carried out using different concentrations of [^3^H]Ins(1,4,5)P_3_ as described in Section 2. Nonspecific binding was determined in the presence of 10 *μ*M D-Ins(1,4,5)P_3_. Each value is the mean ± S.E.M for five independent experiments performed in duplicate.

*B*_max_	*K*_*d*_
(fmol/mg protein)	(nM)
427.77 + 56.67	22.09 + 2.34

**Table 2:** Displacement of [^3^H]Ins(1,4,5)P_3_ binding by inositol phosphates and heparin in human platelet membranes. Platelet membranes were prepared as described in Section 2. The assay medium (100 *μ*L) contained 20 nM [^3^H]Ins(1,4,5)P_3_ with various concentrations of competitive substances and approximately 100 *μ*g of protein. Incubations were carried out at 4°C for 10 minutes. IC_50_ values were calculated by using log probit analysis. The values are the mean ± S.E.M. of three different experiments.

Compound	IC_50_
—	(*μ*M or *μg/mL)
D-Ins(1,4,5)P_3_	0.3 ± 0.5
D-Ins(2,4,5)P_3_	1.90 ± 0.68
GPIP_3_	4.97 ± 1.83
L-Ins(1,4,5)P_3_	354.0 ± 37.0
Heparin	36.79 ± 6.0l*

## Abbreviations

*B*_max:_: Maximum number of binding sites
cAMP: Cyclic adenosine monophosphate
EDTA: Ethylene diamine *N*′, *N*′, *N*′, *N*′-tetraacetic acid
GPIP2: L-*α*-glycerophosphoinositol 4,5-bisphosphate
*K*_*d*_: Apparent dissociation constant
Ins(1,4,5)P_3_: Inositol (1,4,5) triphosphate
PKA: Protein kinase A
PI: Phosphatidylinositol
PKC: Protein kinase C
